# Effects of acne severity and acne-related quality of life on depressive symptoms among adolescents and young adults: a cross-sectional study in Bangladesh

**DOI:** 10.3389/fpsyg.2023.1153101

**Published:** 2023-07-24

**Authors:** Takfi Tasneem, Afroza Begum, Mohammad Rocky Khan Chowdhury, Syed Rahman, Gloria Macassa, Jasmin Manzoor, Mamunur Rashid

**Affiliations:** ^1^International Centre for Diarrheal Disease Research (ICDDR, B), Dhaka, Bangladesh; ^2^Department of Maternal and Child Health, National Institute of Preventive and Social Medicine (NIPSOM), Dhaka, Bangladesh; ^3^Department of Epidemiology and Preventive Medicine, School of Public Health and Preventive Medicine, Faculty of Medicine, Nursing and Health Sciences, Monash University, Melbourne, VIC, Australia; ^4^Division of Insurance Medicine, Department of Clinical Neuroscience, Karolinska Institute, Stockholm, Sweden; ^5^Department of Public Health and Sports Science, Faculty of Health and Occupational Studies, University of Gävle, Gävle, Sweden; ^6^Department of Dermatology, Evercare Hospital Dhaka, Dhaka, Bangladesh

**Keywords:** acne severity, acne-related quality of life, adolescents, young adults, depression

## Abstract

**Introduction:**

Depression is a common mental health disorder and one of the major causes of disability. This study aimed at investigating the relationship of acne severity and acne-related quality of life with depressive symptoms, and the mediating effect of acne-related quality of life in a relationship between acne severity and depressive symptoms.

**Methods:**

This is a cross-sectional study. Data were collected from acne patients attending a tertiary-level hospital, using a questionnaire that comprised three validated instruments – Investigator’s Global Assessment (a single item) scale, Cardiff Acne Disability Index, and Beck Depression Inventory for measuring acne severity, acne-related quality of life, and depressive symptoms, respectively. Logistic regression and linear regression were used to examine the association between acne severity and depressive symptoms and a correlation between the acne-related quality of life and depressive symptoms, respectively. A mediation analysis was also performed to see the mediation effects of acne-related quality of life in a relationship between acne severity and depressive symptoms.

**Results:**

A total of 185 acne patients (155 females, 83.8%) with a mean age was 22.55 ± 8.67 years were included in the study. Adolescents and young adults with severe and moderate acne had 6.14-and 2.28 times higher odds of depression compared to their peers with mild acne, respectively. Patients with low levels of acne-related quality of life had a higher level of depressive symptoms (*β* = 0.42, *p* < 0.001). The total effect (direct + indirect) was also significant (*β* = 0.27, 95% CI: 1.29–4.09), implying the effect of acne severity on depression.

**Conclusion:**

The present study suggests that acne severity and acne-related quality of life were associated with depressive symptoms among patients with acne vulgaris. The study also indicates that the relationship between acne severity and depressive symptoms might occur through a chain-mediating effect of acne disability in this population.

## Introduction

Depression is a common mental health disorder and a major contributor to the global burden of disease (James et al., 2018). More than 264 million people are suffering from depression globally and among them, 13.4% are children and adolescents, 5% are adults and 5.7% are older adults aged 60 and over (Polanczyk et al., 2015; WHO, 2021). Depression is considered a complex condition with many different symptoms and causes (NIH, 2018). A variety of underlying factors, such as biogenic amine deficiency, genetic, environmental, immunologic, endocrine, and neurogenesis, have been identified as mechanisms of the pathophysiology of depression. For example, environmental stressors and heritable genetic factors are thought to trigger immunologic and endocrine responses, which result in structural and functional changes in multiple brain regions, ultimately leading to dysfunctional neurogenesis and neurotransmission that could manifest a constellation of symptoms, which could cause depression (Webster, 2005; Tan et al., 2018). Depression can lead to a variety of psychological and physiological problems that can contribute to decreasing one’s ability to function at work and at home (APA, 2020). Further, depression is common among adolescents and young adults (Venkataraman et al., 2019). Depression increases the risk of suicide, poor health, unhealthy personal behavior, poor social well-being, and lower educational attainment (Anjum et al., 2019; Shukla et al., 2019; Khan et al., 2020). Mental disorders in most cases are treatable and preventable, although more than 75% of people in low-and middle-income countries remain untreated (WHO, 2021).

Depression or depressive symptoms among adolescents has become a neglected public health problem in Bangladesh. Adolescents comprise more than 10% of the total population in Bangladesh. Suicidal behavior (e.g., suicidal ideation, suicidal ideation with a plan, attempted suicide), anxiety, loneliness, lack of close friends, and substance use (e.g., tobacco use, alcohol, marijuana, multiple substances) are highly prevalent among them (WHO, 2017). There is an array of factors that can cause depression ranging from socioeconomic, environmental, and cultural to psychological traits such as stress in a relationship, family and peer pressure, high expectations, lack of financial support and hardships, sleep deficiency, future worry about future, loneliness, longer screen time, toxic psychological environment, academic pressure, workload, size, and so on (Brenneisen Mayer et al., 2016; Abdel Wahed and Hassan, 2017; Saeed et al., 2017; Silva and Figueiredo-Braga, 2018; Al Mamun and Griffiths, 2019; Mridha et al., 2021). Similarly, a stressful situation due to acne severity, as this influences psychological status (Behnam et al., 2013) may trigger hormonal changes in the body that may cause depression among adolescents and young adults.

Acne vulgaris is a chronic inflammatory skin disorder. The pathophysiology of acne is characterized by the overproduction of sebum, abnormal keratinization of hair follicles, and the proliferation of *Propionibacterium acnes* within the pilosebaceous unit. An increased level of androgens at the onset of puberty can stimulate the production of high sebum changes in skin cell activity, inflammation, and colonization of the hair follicles by a bacteria known as *Propionibacterium acnes*, which, in turn, could lead to acne (Walsh, 2009; Jesulola et al., 2018). Acne vulgaris can severely affect psychological functioning and especially may cause anger, anxiety, and depression among adolescents and young adults (Alsulaimani et al., 2020). It is a common chronic inflammatory skin disease that usually develops on the face, chest, shoulders, and back. Acne usually starts in adolescence and may extend up to adulthood (Anon, 2013). It can be classified as a non-inflammatory type characterized by comedones and an inflammatory type characterized by papules, pustules, nodules, and cysts (Anon, 2018). It is estimated to affect 9.4% of the global population, making it the eighth-most prevalent disease worldwide (Tan and Bhate, 2015). Globally, the estimated prevalence of acne varies from 28.9 to 91.3% among adolescents (Asai et al., 2016). In Asian countries such as China, Malaysia, and Saudi, acne vulgaris among adolescents was recorded at over 33, 34, and 56%, respectively (Shen et al., 2012; Muthupalaniappen et al., 2014; Alanazi et al., 2018). Among African countries, Nigeria and Egypt, more than 60% of female adolescents are suffering from acne vulgaris (Okoro et al., 2016; El-Hamd et al., 2017). The probable risk factors of acne vulgaris identified in previous studies were socioeconomic conditions such as dietary factors, topical greasy preparations that block skin pores, humid climate, smoking, obesity, stress, picking, and bacterial infections along with genetic and hormonal (Mahapatra, 2016; Heng and Chew, 2020).

Earlier evidence has shown that psychological issues such as anxiety and depression are found to be more prevalent among acne patients (Asad et al., 2002; Darwish and Al-Rubaya, 2013; Khan, 2017). Acne vulgaris can severely affect mental health, especially depression, obsessive-compulsiveness, and sometimes suicidal ideation (Alsulaimani et al., 2020; Lukaviciute et al., 2020). Previous studies were conducted on the relationship between acne severity and depression in different settings (Asad et al., 2002; Darwish and Al-Rubaya, 2013; Sultana, 2017; Ahmed et al., 2019). For example, the severity of acne is positively related to depression, and also acne vulgaris significantly correlated with the quality of life, i.e., deals with daily social and emotional things based on varying degrees of acne severity (Sultana, 2017; Ahmed et al., 2019). A recent meta-analytic review (Samuels et al., 2020) demonstrated that acne severity, depression and anxiety are associated, but there is an inconsistency in the included studies between acne-related exposures and outcome ascertainment. The review also concluded that most of the studies had a paucity of suitable designs, measurements, and data analyses. Hence, more research is needed to examine the association of acne severity with depression or depressive symptoms. Moreover, to our knowledge, no study has yet examined the mediation effect of acne-related quality of life on the association of acne severity with depressive symptoms, particularly in Bangladesh. Therefore, this study aimed to investigate (i) the relationship of acne severity with depressive symptoms, (ii) a correlation between acne-related quality of life and depressive symptoms, and (iii) whether acne-related quality of life mediates the relationship between acne severity and depressive symptoms.

## Methods

### Study design

A cross-sectional study was conducted among adolescents and young adults recruited from a tertiary hospital in the Department of Dermatology and Venereology, Evercare Hospital Dhaka.

### Selection of participants

Participants for this study were collected from outpatient visits in the Department of Dermatology and Venereology, Evercare Hospital Dhaka, Bangladesh. Patients who visited dermatologists for acne vulgaris care in the hospital were randomly selected for the participation. Patients were invited to take part in the study if they met the following inclusion criteria: (i) aged between 12 and 40 years of either sex, (ii) having a diagnosis of acne vulgaris during the period between 21st April 2018 and 21st June 2018, and (iii) attended the outpatient care at the department of dermatology. However, patients with other dermatological diseases/disorders such as Rosacea, Hives, Measles, Lupus, and Melasma were excluded, and no repeated visits were considered in the study. In the data collection process, a dermatologist (JM) and a general physician (TT) checked the patient’s history according to the inclusion criteria. If the patients matched the criteria, they were invited to fill in a survey questionnaire with their consent.

A self-administered questionnaire comprised of three validated instruments – Investigator’s Global Assessment (IGA) a single item scale, Cardiff Acne Disability Index (CADI), and Beck Depression Inventory (BDI) for measuring acne severity, acne-related quality of life, and depressive symptoms, respectively – was used to collect data in this study. To facilitate patients’ comprehension, all the instruments – IGA, CADI, and BDI – were translated into Bengali. The translation was made by a professional translator who had full professional language proficiency in both Bengali and English. Afterward, the translated instruments were tested on a sample of 10 individuals, comprising both males and females, to confirm whether the questions incorporated in the instruments were well understood. In addition, a set of demographic variables such as age, gender, education, marital status, occupation, and income was included in the questionnaire.

Two hundred outpatients were invited by the hospital to take part in the study. Out of 200, a total of 185 completed the survey questionnaire with their informed consent, giving a response rate of 93%. Further, a data collection process, such as filling in the questionnaire and entering the data into the MS-Excel system, was initiated for the patients who agreed to take part in the study, led by an employee in the hospital administration.

### Sample size calculation

Based on a proportion of acne severity in a previous study, a sample size calculation was conducted considering a statistical power of 80%, and an acceptable alpha error rate of 5% using Epi info software version 7.2. A minimum of 150 participants was required for this study. Eventually, a total of 185 outpatient patients fulfilled the inclusion criteria and agreed to participate in this study.

### Measures

#### Acne severity

Acne severity was measured using the IGA (Langley et al., 2015) scale in this study. Only acne on the face was considered in the study; however, acne on the chest, back, or shoulders were not considered. The scale consists of a single item where respondents responded on a 5-point Likert scale (0 = almost clear; 1 = a few scattered comedones and a few small papules; 2 = mild and easily recognizable, less than half the face is involved where some comedones and some papules and pustules; 3 = moderate, more than half the face is involved where many comedones, papules, and pustules; 4 = severe, entire face is involved which covered with comedones, numerous papules and pustules, and few nodules and cysts). This scale has been further simplified for dermatologists who used the scale for grading acne severity as 0–2 = mild, 3 = moderate, and 4 = severe (Alsulaimani et al., 2020).

#### Acne-related quality of life

Acne-related quality of life was assessed using the CADI (Motley and Finlay, 1992). The CADI is an instrument that assesses the effect of acne on quality of life, which consists of five-item. The first two-item addresses the psychological and social consequences of acne in general; the third one targets those with acne in exposed areas; the fourth one enquires into the patient’s psychological state, and the last one asks for the patient’s subjective assessment of current acne severity. Respondents answered on a 4-point Likert scale ranging from 0 = not at all to 3 = severely; a higher score indicates a lower level of acne-related quality of life. All items were summed to generate a total CADI score, which is ranging from 0 to 15 points where higher values represent a lower level of acne-related quality of life. Moreover, consideration of a conceptual rather than literal approach in our translation process guaranteed the usage of specific words and phrases reflecting the Bangladeshi culture. The reliability of the scale was measured using Cronbach’s α in the present study, which was 0.74, indicating an adequate internal consistency. An earlier study also checked internal consistency of a Persian version of CADI was shown almost similar Cronbach’s *α* (0.79) (Aghaei et al., 2006).

#### Depression

Self-reported depressive symptoms was assessed using the BDI scale (Jackson-Koku, 2016) in this study, which consists of 21-item. Respondents answered each item rated on a 4-point Likert (0 to 3); a total score on the scale was ranging from 0 to 63; higher scores indicate a high level of depressive symptoms. The level of depressive symptoms was also divided as normal (1–10), mild (11–16), Moderate (17–30), and Severe (31 and above). The variable was categorized as no (normal) and yes (mild to severe) in the present study. The reliability of the scale measured using Cronbach’s α was 0.79 in the present study, which is indicating an adequate internal consistency. A previous study (Mostafa Alim et al., 2020) measured validation of a Bengali version of BDI (21-item) among Bengali population using Cronbach’s α, which was 0.84 in average of all items.

#### Socio-demographic characteristics

Socio-demographic characteristics considered for this study were: *age* – adolescents and young adults aged between 12 and 40; *gender* was categorized into female and male; *education* was divided into secondary or below, higher secondary, and bachelor’s degree or above; *marital status* – married and unmarried; *occupation* was classified as student, job holder, business, and homemaker, and monthly *family income* was represented in dollar in this study.

### Statistical analysis

A descriptive analysis was performed to compute frequencies and proportions for categorical data as well as the mean and standard deviation for continuous data. The difference in the prevalence of depression by background variables was tested with a chi-squared test, and the mean difference was evaluated by an independent *t*-test. Variables that appeared significant in bivariate analysis were controlled for in the multivariate regression model. A logistic regression was performed to examine the association of acne severity with depressive symptoms (the outcome was treated as dichotomized – yes or no), as well as a linear regression model was fitted to see a correlation between the acne-related quality of life and depressive symptoms (the outcome was treated as a continuous variable). The significance level was set at *p* < 0.05 with a 95% confidence interval (CI). All analyses were performed using IBM SPSS Statistics (version 27).

**Figure 1:**
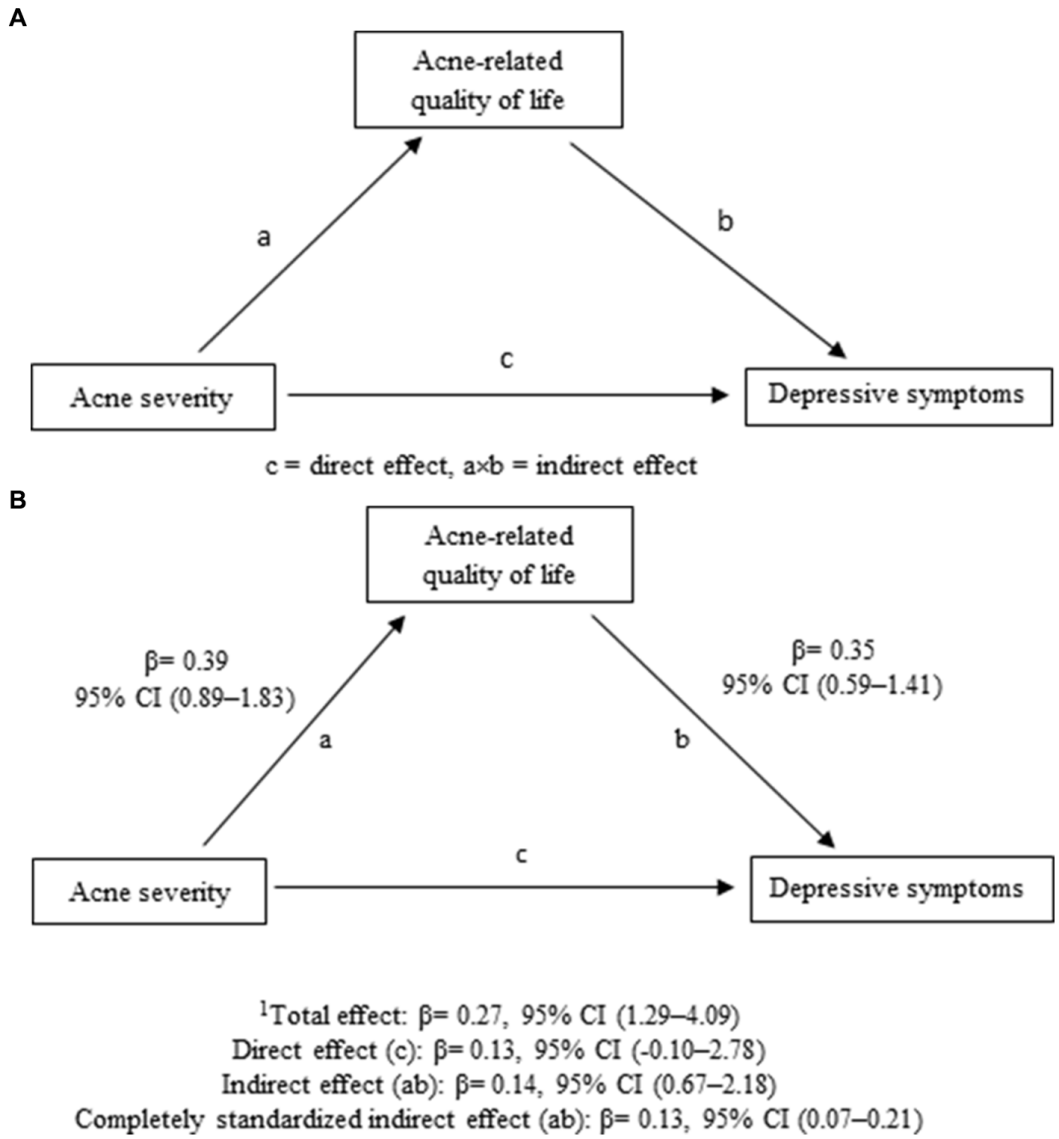
**(A)** Path diagram showing acne disability as a mediator in acne severity and depressive symptoms association. **(B)** Path diagram showing acne-related quality of life as mediator in acne severity and depressive symptoms association, indicating standardized *β* in direct, indirect, and total effect; ^1^Total effect = (indirect + direct); CI, Confidence Interval.

For mediation analysis, PROCESS by Andrew F. Hayes (version 3.5) (Hayes, 2018) macro for SPSS was followed to see the indirect effect of exposure on the outcome, which was defined as the product of path a and b, i.e., a × b (Figure 1A). Mediation analysis decomposes the “total” effect of exposure (acne severity) on the outcome (depressive symptoms) into “direct” and “indirect” effects. Direct effect was calculated between acne severity and depressive symptoms. Indirect effect was comprised of a combined effect of path a and b – i.e., path a between acne severity and acne-related quality of life, and path b between acne-related quality of life and depressive symptoms (Figure 1A). To identify the upper and lower bounds of a 95% CI for the direct, indirect, and total effect, a bias-corrected 5,000 bootstrapped CI was generated by PROCESS. Statistical significance result was regarded when zero was not included between the lower and upper limit of the 95% CI.

## Results

Background characteristics are presented in Table 1. The mean age of the participants was 22.55 years (SD ± 8.68). More than 80% of the participants were female, and around 25% were married. More than 50% of the participants were student, and approximately 50% had a bachelor’s degree or above. Around 20% of the participants had job or business. More than 50% of the participants had yearly family income more than 100,000 BDT and around 14% were 300,000 or above. Approximately 70% was seeking any kind of acne treatment before treating in the Department of dermatology. Approximately 43% of the participants had severe acne, and the prevalence of depressive symptoms (mild to severe) was 65%.

**Table 1:** Background characteristics of the participants.

Variables	Frequency (%)
Age (years) (*M*, SD)	22.55 ± 8.68
Gender
Female	155 (83.8)
Male	30 (16.2)
Education
Secondary or below	47 (25.4)
Higher Secondary	48 (25.9)
Bachelor’s degree or above	90 (48.7)
Marital status
Unmarried	139 (75.1)
Married	46 (24.9)
Occupation
Student	124 (67.0)
Job holder	25 (13.5)
Business	11 (6.0)
Homemaker	25 (13.5)
Family income (in Dollar)
Up to 1,175	102 (55.1)
1,176–2,350	45 (24.3)
2,351–3,525	12 (6.5)
3,526 or above	26 (14.1)
Acne severity
Mild	39 (21.1)
Moderate	67 (36.2)
Severe	79 (42.7)
Depression^1^
No	64 (34.6)
Yes	121 (65.4)
Depressive symptoms^1^ (*M*, SD)	14.50 ± 7.70
Acne disability (*M*, SD)	8.86 ± 2.69

^1^Depression in categorical scale (yes = with depression, no = without depression), continuous scale (range: 0–39); M, mean; SD, standard deviation.

Table 2 shows the characteristics of the participants with or without depressive symptoms by background characteristics. Age and occupation appeared to be significantly different between those who were with depressive symptoms and without depressive symptoms. A total of 65% of participants (aged M ± SD: 22.02 ± 4.95) reported they had depressive symptoms whereas 35% (aged M ± SD: 21.98 ± 6.39) reported no depressive symptom. In terms of occupation, 65% of students appeared with depressive symptoms and 35% with no depressive symptoms. Among homemaker, 84% were experienced depressive symptoms and 16% with no depressive symptoms.

**Table 2:** Characteristics of participants with or without depressive symptoms across the background variables (*n* = 185).

Variables	Depressive symptoms^1^		Value of *p*
	Yes, *n* (%)	No, *n* (%)	
Age (years) (*M*, SD)	121 (22.02 ± 4.95)	64 (21.98 ± 6.39)	0.01
*Gender*	0.13
Female		105 (67.7)	50 (32.3)
Male		16 (53.3)	14 (46.7)
*Education*	0.56
Secondary or below		29 (61.7)	18 (38.3)
Higher secondary		33 (68.8)	15 (31.3)
Bachelor’s degree or above		59 (65.6)	31 (34.4)
*Marital status*	0.16
Unmarried		87 (62.6)	52 (37.4)
Married		34 (73.9)	12 (26.1)
*Occupation*	0.05
Student		80 (64.5)	44 (35.5)
Job holder		12 (48.0)	13 (52.0)
Business		8 (72.7)	3 (27.3)
Homemaker		21 (84.0)	4 (16.0)
*Family income (in Dollar)*	0.52
Up to 1175		69 (67.6)	33 (32.4)
1,176–2,350		31 (68.9)	14 (31.1)
2,351–3,525		7 (58.3)	5 (41.7)
3,526 or above		14 (53.8)	12 (46.2)

^1^Depressive symptoms, yes = with depressive symptoms and no = without depressive symptoms; M, mean; SD, standard deviation.

Table 3 represents the results from the multiple logistic regression analysis. In the adjusted analysis, the odds of moderate acne severity (OR: 2.28, 95% CI: 1.02–5.14) and severe acne severity (OR: 6.14, 95% CI: 2.62–14.38) were 2.28 and 6.14 times, respectively, higher of having depressive symptoms among adolescents and young adults when compared with mild acne severity.

**Table 3:** Multiple logistic regression analyses between acne severity and depressive symptoms.^1^

Variable	Unadjusted analysis			Adjusted analysis^2^		
	OR	95% CI	Value of *p*	OR	95% CI	Value of *p*
Acne severity
Mild	1			1		
Moderate	2.27	(1.01–5.07)	0.05	2.28	(1.02–5.14)	0.04
Severe	6.13	(2.62–14.36)	<0.001	6.14	(2.62–14.38)	<0.001
Age				1.01	(0.93–1.09)	0.91
Occupation				0.95	(0.66–1.37)	0.79

^1^Depressive symptoms was assessed using the BDI scale and treated here as categorical scale (yes, with depression; no, without depression); ^2^Age and participants’ occupations were controlled for in the adjusted analysis; OR, odds ratio; CI, confidence interval.

Table 4 shows that patients with a low level of acne-related quality of life had a higher level of depressive symptoms in both unadjusted and adjusted analyses (*β* = 0.42, *p* < 0.001). The regression model was statistically significant (*p* < 0.001), and 36% of the total variation was explained by the model.

**Table 4:** Multiple linear regression analyses between acne disability and depressive symptoms.^1^

Variable	Unadjusted analysis			Adjusted analysis^3^		
	*β*	SE	Value of *p*	*β*	SE	Value of *p*
Acne-related quality of life^2^	0.40	0.19	<0.001	0.42	0.20	<0.001
Age				−0.07	0.12	0.39
Occupation				0.16	0.58	0.06
*R^2^*	0.36			0.39		
Adjusted *R*^2^	0.35			0.36		

^1^Depressive symptoms was assessed using the BDI scale and treated here as continuous scale (range: 0–39) where higher scores represent a greater level of depression; ^2^acne-related quality of life was assessed using the CADI scale – the total score of the scale is ranging from 0 to 15 points where higher values represent a lower level of acne-related quality of life; ^3^age and participants’ occupations were controlled for in the adjusted analysis; β, standardized regression coefficient; SE, standard error.

As predicted, the relationship between acne severity and depressive symptoms could positively be mediated by the acne-related quality of life (Figure 1A). As seen in Figure 1B, the indirect effect was found to be significant (*β* = 0.14, 95% CI: 0.07–0.21), which might be regarded as reporting a mediation effect – indicating that the relationship between acne severity and depressive symptoms was positively related only through the acne-related quality of life. The total effect was also shown as a significant result (*β* = 0.27, 95% CI: 1.29–4.09). However, the direct effect of acne severity on depressive symptoms was not statistically significant (*β* = 0.13, 95% CI: −0.10–2.78).

## Discussion

The present study evaluated the relationship between acne severity, acne-related quality of life, and depressive symptoms. The results revealed that depressive symptoms were negatively influenced by moderate and severe levels of acne severity, and indicated also a positive correlation between the level of acne-related quality of life and depressive symptoms among adolescents and young adults. Moreover, this study found a mediation effect of acne-related quality of life in the relationship between acne severity and depressive symptoms in this study population.

The present findings highlighted a higher prevalence of acne in females (84%) outnumbering males (16%). Previous studies in Nepal, India, Pakistan, Saudi Arabia, and Egypt also revealed that acne was more common in females as compared to males (Alsulaimani et al., 2020; Dhoot et al., 2020; Tayel et al., 2020; Naveed et al., 2021). In our study, there was a preponderance of severe acne in almost 43% of the patients; however, some studies reported a higher prevalence of mild acne (Alsulaimani et al., 2020; Tayel et al., 2020; Heng et al., 2021). The prevalence of depression (65%) was higher in acne patients in comparison to preceding studies (Ahmed et al., 2019; Lukaviciute et al., 2020).

The obtained result regarding the association of acne severity, either moderate or severe, with depressive symptoms was in line with previous findings, indicating that the level of acne severity was associated with adverse psychological impact and increased psychological symptoms such as depression that has an adverse impact on the lives of adolescents and young adults (Gupta and Gupta, 1998; Uhlenhake et al., 2010; Ahmed et al., 2019). In contrast, some previous studies did not find an association between acne severity and psychological symptoms like anxiety, depression, and stress (Aktan et al., 2000; Yolaç Yarpuz et al., 2008). A possible reason for this contradictory result could be that individuals with depression in the previous studies might not be caused by acne severity (Golchai et al., 2010), rather they were experiencing depression due to other reasons, e.g., stress, mood changes, and physical illness (Wahid et al., 2021), which were not taken into account in the present study. Further, adolescents’ quality of life from all dimensions of life, e.g., stress from family and other forms of social demand, could enhance psychological burden, e.g., depression (Naz et al., 2020). The findings from the present study indicate that acne-related quality of life was also positively connected to depression. This finding is supported by a previous study showing that mild acne vulgaris has a mild impact on quality of life (Ogedegbe and Henshaw, 2014). This finding may suggest that the presence of acne creates problems with patients’ quality of life that may cause mental illnesses such as depression. This may reflect the fact that a patient’s acne-related quality of life should be improved rather than only improvement of acne severity (Martin et al., 2001; Ammad et al., 2008).

The result from the present study also indicates that acne-related quality of life had a mediation effect on the relationship between acne severity and depressive symptoms. This finding suggests that besides clinical treatment it would paralleling be more effective in propelling acne-related quality of life to treat depression among adolescents and young adult patients with acne vulgaris (Tayel et al., 2020; Kaikati et al., 2021). Given the concern of depression due to acne, it is critical that physicians monitor the mood symptoms of patients with acne and begin early depression management or seek consultation from a psychiatrist when needed. A descriptive study conducted in Bangladesh demonstrated that evaluation of the acne-related quality of life is more effective and helpful for better psychological treatment and management in adolescent and adult patients with acne (Rahman et al., 2022).

### Strengths and limitations

The strengths of this study are the relatively high response rate and the fact that the diagnosis and severity classification of the patient’s acne was performed by an expert dermatologist and used validated instruments for all measurements. To our best knowledge, this is the first study to examine the mediation effect of acne-related quality of life in the relationship between acne severity and depression, although previous studies investigated the association of acne severity and acne-related quality of life with depression in different settings. There are several limitations of this study that should be considered. One of the potential limitations is that Type II error could occur in this study due to a small sample size, which could decrease the reliability of the study. Similarly, the probability of Type I error could also occur because of the significance levels of hypothesis test was chosen. Having a dichotomized variable of depression in this study could lead to a loss of information, which could impact the accuracy of the analysis and increase the risk of a positive result being a false positive. Moreover, due to a small sample size, we did not consider all potential confounding variables in this study. Another potential limitation is that use of self-reported measure that may fail to reflect the actual clinical condition of the patients, which could lead to common method bias in the results. Depression might already be an existing condition due to other reasons that did not investigate in this study. Because the survey was cross-sectional, a cause-and-effect relationship could not be drawn for this study. This study was conducted in only one tertiary private hospital where most affluent patients usually attend this hospital and most of the patients have a particular socio-economic background. Therefore, before generalizing the present findings, further research is warranted taking a larger sample from hospitals at different levels such as private and public.

### Practical implications

The present study indicated that acne-related quality of life should pay attention to enhancing the coping strategy of patients with acne severity, either moderate or severe, to making positive attitudes that could lead to lessening the onset of depression. Simultaneously, depressive symptoms among acne vulgaris patients can also be alleviated by promoting the relief of acne-related quality of life, rather than only focusing on acne severity. To prevent the development of negative psychology, medical staff and public health practitioners should encourage and unite family members, friends, and community members to provide comprehensive support to patients, which may contribute to alleviating depression. The findings of this study may also be used for planning and implementing interventions to improve and promote the quality of life of patients with acne. Moreover, psychotherapists could support patients on how to deal with mental stress such as anxiety and depression due to acne severity.

## Conclusion

Our findings suggest that acne severity and acne-related quality of life were positively associated with depressive symptoms among adolescents and young adult patients with acne vulgaris. The study has also confirmed that acne severity may not directly relate to depressive symptoms in this population, rather the relationship might occur through a chain mediating effect of acne-related quality of life. This research has practical guidance for dermatologists to provide a more holistic management approach to treat acne vulgaris whilst the patients are getting used to suffering from depressive symptoms due to acne vulgaris.

## Data availability statement

The raw data supporting the conclusions of this article will be made available by the authors, without undue reservation.

## Ethics statement

The studies involving human participants were reviewed and approved by Bangladesh University of Health Sciences and the Hospital (BUHS/BIO/EA/17/116). The patients/participants provided their written informed consent to participate in this study.

## Author contributions

MR co-developed the study design, performed the data analyses, and drafted the manuscript. TT and AB performed the data collection and data processing, and drafted the manuscript. MC and SR co-developed the study design, contributed to the analyses, and reviewed the initial drafts of the manuscript. GM co-developed the study design and reviewed the initial drafts of the manuscript. JM performed the data collection and data processing. All authors have read and approved the final manuscript.

## Conflict of interest

The authors declare that the research was conducted in the absence of any commercial or financial relationships that could be construed as a potential conflict of interest.

## Publisher’s note

All claims expressed in this article are solely those of the authors and do not necessarily represent those of their affiliated organizations, or those of the publisher, the editors and the reviewers. Any product that may be evaluated in this article, or claim that may be made by its manufacturer, is not guaranteed or endorsed by the publisher.
